# The prevalence of growth hormone deficiency in survivors of subarachnoid haemorrhage: results from a large single centre study

**DOI:** 10.1007/s11102-017-0825-7

**Published:** 2017-08-18

**Authors:** Sumithra Giritharan, Joanna Cox, Calvin J. Heal, David Hughes, Kanna Gnanalingham, Tara Kearney

**Affiliations:** 1 Department of Endocrinology, Salford Royal NHS Foundation Trust, Stott Lane, Salford, Greater Manchester, M6 8HD UK; 2 Vascular Research Network, Salford Royal NHS Foundation Trust, Stott Lane, Salford, Greater Manchester, M6 8HD UK; 3 Centre for Biostatistics, Faculty of Biology, Medicine and Health, Manchester Academic Health Science Centre, University of Manchester, Manchester, UK; 4 Department of Neuroradiology, Salford Royal NHS Foundation Trust, Stott Lane, Salford, Greater Manchester, M6 8HD UK; 5 Department of Neurosurgery, Salford Royal NHS Foundation Trust, Stott Lane, Salford, Greater Manchester, M6 8HD UK; 6 Manchester Academic Health Sciences Centre (MAHSC), University of Manchester, Manchester, UK; 7 Department of Endocrinology and Diabetes, Salford Royal NHS Foundation Trust, Stott Lane, Salford, Greater Manchester, M6 8HD UK

**Keywords:** Subarachnoid haemorrhage, Hypopituitarism, Growth hormone deficiency

## Abstract

**Objective:**

The variation in reported prevalence of growth hormone deficiency (GHD) post subarachnoid haemorrhage (SAH) is mainly due to methodological heterogeneity. We report on the prevalence of GHD in a large cohort of patients following SAH, when dynamic and confirmatory pituitary hormone testing methods are systematically employed.

**Design:**

In this cross-sectional study, pituitary function was assessed in 100 patients following SAH. Baseline pituitary hormonal profile measurement and glucagon stimulation testing (GST) was carried out in all patients. Isolated GHD was confirmed with an Arginine stimulation test and ACTH deficiency was confirmed with a short synacthen test.

**Results:**

The prevalence of hypopituitarism in our cohort was 19% and the prevalence of GHD was 14%. There was no association between GHD and the clinical or radiological severity of SAH at presentation, treatment modality, age, or occurrence of vasospasm. There were statistically significant differences in terms of Glasgow Outcome Scale (GOS; p = 0.03) between patients diagnosed with GHD and those without. Significant inverse correlations between GH peak on GST with body mass index (BMI) and waist hip ratio (WHR) was also noted (p < 0.0001 and p < 0.0001 respectively).

**Conclusion:**

Using the current testing protocol, the prevalence of GHD detected in our cohort was 14%. It is unclear if the BMI and WHR difference observed is truly due to GHD or confounded by the endocrine tests used in this protocol. There is possibly an association between the development of GHD and worse GOS score. Routine endocrine screening of all SAH survivors with dynamic tests is time consuming and may subject many patients to unnecessary side-effects. Furthermore the degree of clinical benefit derived from growth hormone replacement in this patient group, remains unclear. Increased understanding of the most appropriate testing methodology in this patient group and more importantly which SAH survivors would derive most benefit from GHD screening is required.

## Introduction

Subarachnoid Haemorrhage (SAH) is a rare but devastating event that occurs in about 8 to 10 per 100,000 patients per year [1]. In the past mortality was approximately 50% and about a third of survivors did not regain full independence [2]. Improvements in neurointensive care and the introduction of endovascular procedures have improved survival rates, with case fatality decreasing by 17% in absolute terms in the past three decades [3]. However, this improvement in mortality has unmasked the long term consequences of this life changing event. With increasing interest in patient reported quality of life as an outcome marker in the treatment of chronic diseases it is now clear that in spite of good physical and neurological outcome, a significant proportion of survivors report impaired quality of life [4–7].

Chronic sequelae of SAH include poor memory, fatigue, anxiety, depression and impaired quality of life [4, 8–11]. Undoubtedly some of the cognitive, emotional and psychosocial consequences seen in survivors of SAH resemble that of patients with untreated hypopituitarism [2], specifically Growth Hormone Deficiency (GHD). Pituitary dysfunction developing post SAH was first documented in the seminal publication of Kelly et al. [12]. Early studies have reported the prevalence of hypopituitarism in SAH survivors to be as high as 55% [2, 13–16]. However, more recent studies report a much lower prevalence of pituitary dysfunction in this patient group [17–22] (Table 1). A recent meta-analysis has demonstrated that the pooled frequency of long term GHD is 19%, however the range reported in the literature is wide, between 0 and 37% [23].

**Table 1:** Summary of studies investigating hypopituitarism after subarachnoid haemorrhage

Study	Biochemical test	Number of patients	Time of assessment	Prevalence of hypopituitarism	Prevalence of GHD
Kreitschmann-Andermahr et al. [2]	Basal hormone values, ITT, TRH-LHRH-arginine test	40	27.3 months (mean)	55%	20%
Aimaretti et al. [16]	Basal hormone values, GHRH-arginine test, morning serum cortisol, 24 urinary cortisol	40	3 months	37.5%	25%
Aimaretti et al. [39]	Basal hormone values, GHRH-arginine test, morning serum cortisol, 24 urinary cortisol	32	12 months	37.5%	21.8%
Dimopoulou et al. [15]	Basal hormone values, IGF-1 level, low dose ACTH test	30	12–24months	47%	37%
Tanriverdi et al. [14]	Basal Hormone values (within 24 h) GST, GHRH-arginine (12 months)	22	Within 24 h 12 months	63.6% 45%	22.7% 36.4%
Jovanovic et al. [13]	Basal hormone values, IGF-1	93	1.8 years (mean)	49.5%	29%
Klose et al. [21]	ITT, SST, GHRH-arginine test, clomiphene test	62	14 months	0%	0%
Parenti et al. [33]	Basal hormone values, IGF-1 levels	60	Within 72 h	56.9%	22%
Lammert et al. [17]	Basal hormone values, SST, ITT*	20	12 months	15%	15%
Dutta et al. [34]^Ŧ^	Basal hormone values, IGF-1 level	60	At or after 6 months	31.6%	15%
Karaca et al. [29]	Basal hormone values, GST	20	3 years	20%	20%*
Gardner et al. [19]	GST, SST, GHRH-arginine	64 50	3 months 12 months	45% 12%	20% 10%
Hannon et al. [20]	ITT, GST, SST	41	15 months	14.6%	13.3% (4/30)
Khajeh et al. [22] (HIPS)	Basal Hormone Values, Ghrelin test (Baseline) +/− metyrapone stimulation test Baseline hormone values and GHRH-arginine (6 months) Basal hormone values and GHRH-arginine test (14 months)	84 (baseline) 72 68	32 days (mean) 6 months 14 months	44% 31% 9%	31% 11% 7%
Kronvall et al. [18]	Basal hormone values, GHRH-arginine test Basal hormone values, GHRH-arginine test, ITT, SST	45 44	3–6 months 12–24 months	27% 43%	7% 25%

To calculate the frequency of pituitary dysfunction at each time point, the actual number of patients at each follow-up time point is used as the denominator, rather than the number of patients at baseline. Studies providing pooled traumatic brain injury (TBI) and SAH data are not included

*ITT only performed in patients with suspected GHD and ACTH deficiency. 3 and 6 months data from this cohort not presented

^Ŧ^Retrospective and prospective cohort

We present the results from the screening phase of a study to assess the impact of GH replacement in survivors of SAH with GHD. The prevalence of GHD detected in this cohort using the testing protocol employed is reported.

## Subjects and methods

### Patient recruitment

This was a single centre cross sectional study of patients with SAH presenting to the regional neurosurgical centre. Patients who had received treatment for SAH between 2006 and 2014 were invited to participate 1 year or more after ictus. Study posters were also placed in a local head injury centre.

SAH was confirmed by the presence of blood on computed tomographic (CT) imaging of the brain or on cerebrospinal fluid (CSF) analysis obtained by lumbar puncture. Exclusion criteria were clinical contraindication to dynamic pituitary testing, history of cranial radiotherapy, hypothalamic/pituitary disease that was diagnosed prior to SAH and recent use of oral corticosteroids. Participants with prior history of hormonal deficiency were required to have been on stable replacement (where appropriate) for at least 3 months preceding recruitment.

This study was approved by NRES Committee North West – Greater Manchester West (Reference 14/NW/0191). All participants were required to provide written informed consent prior to study enrolment.

### Measures of severity of SAH

The World Federation of Neurosurgical Societies (WFNS) grading system [24] was used to assess clinical severity of patients at presentation based on clinical information from medical records. Fisher grading system [25] was used to grade the radiological severity of SAH. If this information was not clear from the medical notes, the admission neuroimaging was reviewed by a single neuroradiologist. The location of the aneurysm, presence of hydrocephalus, insertion of extraventricular drain (EVD), presence of vasospasm and type of intervention (neurosurgical clipping or endovascular coiling) were recorded. Glasgow Outcome Scale (GOS) [26] was assessed at time of hormonal screening and graded in severity as grade 1 (death), grade 2 (persistent vegetative state), grade 3 (severe disability—conscious but disabled), grade 4 (disabled but independent as far as daily life) and grade 5 (good recovery and there may be minor deficits).

### Anthropometric and quality of life measures

Body weight (measured to the nearest 0.1 kg using a Marsden weighing scale) and height (measured to the closest 0.5 cm) were used to calculate the body mass index (BMI). Waist and hip circumference were measured to the closest 0.5 cm and the waist to hip ratio (WHR) was then calculated. All participants completed the quality of life in adults with growth hormone deficiency (QoL-AGHDA) questionnaire [27] at the screening visit.

### Clinical protocol

All patients agreeing to take part were screened by measurement of baseline pituitary hormones (IGF-1, testosterone/estradiol, LH, FSH, cortisol, ACTH, fT4, TSH and prolactin) and a glucagon stimulation test (GST). Given the tendency of the GST to overestimate ACTH insufficiency, all patients with a suboptimal cortisol response on GST were required to undergo a confirmatory short synacthen test (SST).

All patients with Isolated growth hormone deficiency (IGHD) were required to undergo a second confirmatory test; the arginine stimulation test (AST). Given that this was primarily a study to assess the impact of GH replacement on survivors of SAH with GHD, participants who did not demonstrate impaired quality of life on the QoL-AGHDA questionnaire (and therefore did not meet the National Institute for Health and Care Excellence criteria for GH Replacement [28]), were allowed to decline confirmatory testing of GH axis. Protocol details of the dynamic tests used is provided in the Appendix section.

### Assay and diagnostic criteria

Prior to the 26th January 2015, plasma cortisol, fT4, TSH, prolactin, LH, FSH, testosterone and estradiol were analysed using Electrochemical Luminescent Immunoassay (Roche Cobas 8000). After this time, these measurements were analysed using competitive Chemiluminescent Immunoassay (Siemens Advia Centaur). ACTH and GH were analysed using Siemens Immulite 2000 Two Site Enzymatic Chemiluminescent Immunoassay. IGF-1 levels were analysed using Siemens Immulite 2000 Enzymatic Chemiluminescent Immunoassay.

ACTH deficiency was diagnosed as a failure to reach a peak cortisol value of 450 nmol/L on both the GST and SST. Severe GHD was diagnosed as a failure to reach a peak GH value of 3 µg/L on dynamic testing. Hypogonadotrophic hypogonadism in men was diagnosed if a low serum testosterone (morning sample) was associated with low or inappropriately normal gonadotrophin level. In premenopausal women, hypogonadotrophic hypogonadism was defined as low serum estradiol and inappropriately low gonadotrophins associated with amenorrhoea or oligomenorrhoea. In post-menopausal women, this was defined as inappropriately low gonadotrophins for age. Secondary hypothyroidism was defined as a low serum free T4 associated with low or inappropriately normal serum TSH.

### Statistical analyses

Data analysis was carried out using IBM SPSS Statistics 22 (IBM SPSS Statistics for Windows, Version 22.0 Armonk, NY, USA: 2013). The prevalence of GHD was reported with descriptive statistics. Categorical data was analysed with either the Chi-squared test or the Fisher exact test where appropriate. Non-categorical data was analysed using the t-test or the Mann–Whitney *U* test where appropriate. A two tailed p-value <0.05 was considered statistically significant.

## Results

### Patient demographics and clinical features of subarachnoid haemorrhage

One hundred patients (32 males and 68 females) were screened, with a mean age at screening of 57 ± 10 years (range 32–83 years). The mean age at time of SAH was 53 ± 10 years (range 24–78 years) and the median interval from ictus to pituitary hormone testing was 35 months (range 14–117 months). The mean body weight was 74.6 ± 15.1 kg, with a BMI of 27.3 ± 4.6 and mean WHR of 0.89 ± 0.08. Majority of patients presented with WFNS grades 1 or 2 (n = 82) and the commonest Fisher grade was 4 (n = 39; Table 2).

**Table 2:** Clinical characteristics of SAH survivors included in our cohort

	Number (n = 100)
Male/female	32/68
Mean age at screening (months)	57 ± 10
Mean age at time of SAH (months)	53 ± 10
Median time from SAH to pituitary hormone testing (months)	35 (IQR 22–73)
Mean weight (kg)	74.6 ± 15.1
Mean BMI	27.3 ± 4.6
Mean WHR	0.89 ± 0.08
Procedure	
Endovascular coiling	67
Neurosurgical clipping	15
Multiple coiling procedures during acute admission	4
None	14
Location of aneurysm	
Anterior circulation	
Anterior communicating artery	27
Middle cerebral artery	19
Posterior communicating artery	18
Internal carotid artery	7
Pericallosal artery	1
Posterior circulation	
Basilar artery	6
Posterior inferior cerebellar artery	3
Vertebral artery	1
Perimesencephalic	14
Multiple aneurysms (unable to determine site of bleeding)	4
WFNS	
1	69
2	13
3	4
4	3
5	6
Unavailable	4
Fisher grade*	
1	13
2	15
3	11
4	39
Too late	2
Not available**	6
GOS	
4	15
5	85
QoL-AGHDA ≥11	69

*Patients presenting with perimesencephalic pattern SAH were not given Fisher score

**In these patients copies of admission imaging were not available electronically

On angiographic studies, anterior circulation aneurysms (n = 72) and notably anterior communicating artery aneurysms, (n = 27) were the commonest (Table 2). Presence of other incidental aneurysms (not source of acute bleeding) was noted in 21 (21%) patients. Insertion of EVD was required in 16 (16%) patients due to the development of hydrocephalus. Twenty-three patients had radiological evidence of vasospasm during the acute admission. In one patient, details regarding the acute in patient admission were not available as she was managed at different centre.

Interventional procedures were carried out in 86 (86%) patients and this was either endovascular coiling or surgical clipping. Perimesencephalic type SAH was diagnosed in 14 (14%) patients as no aneurysms were detected on neuroimaging and therefore these patients did not require endovascular or neurosurgical treatment. In four patients, multiple aneurysms were coiled during the acute episode (Table 2).

### Baseline pituitary profile and glucagon stimulation tests

**Fig. 1:**
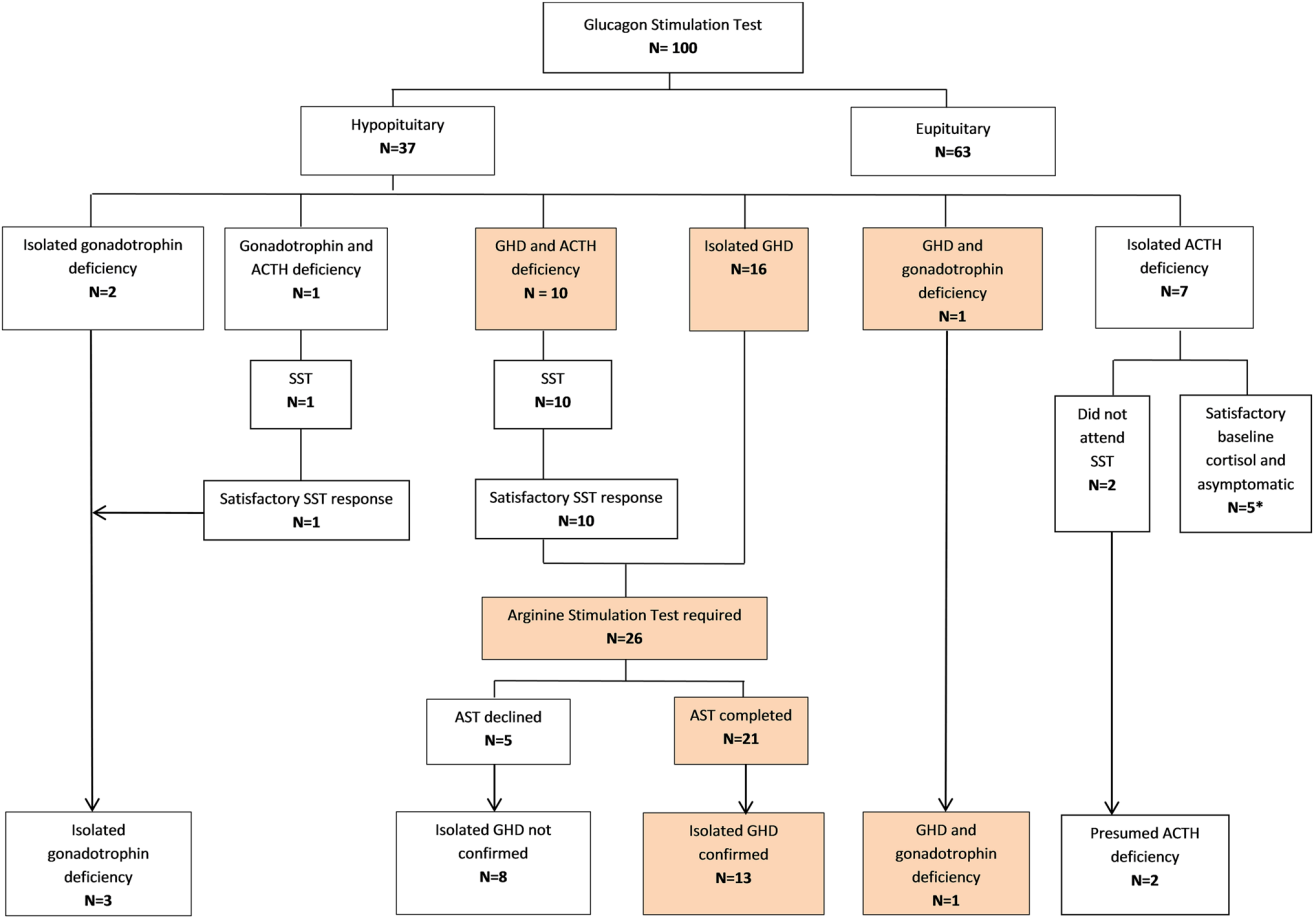
Flowchart demonstrating progression of patients through the study protocol. Only male hypogonadism was detected in this cohort. *Five patients diagnosed with ACTH deficiency on GST, were deemed to be sufficient after results were re-reviewed (see results section)

After screening GST, 37 (37%) patients were diagnosed with some degree of hypopituitarism (Fig. 1). The most common deficiency was severe GHD which was diagnosed in twenty-seven of patients, followed by ACTH deficiency in eighteen patients and lastly gonadotrophin deficiency in four patients. No cases of female hypogonadism were detected. No cases of TSH deficiency or hyperprolactinaemia were detected. In patients with GHD, sixteen patients had isolated GHD, ten patients had GHD in association with ACTH deficiency and one patient had GHD in association with hypogonadism.

### Short synacthen test

Results of all patients with peak cortisol values less than 450 nmol/L on GST were reviewed. One patient had baseline cortisol of 328 nmol/L with a peak cortisol was 425 nmol/L on GST and this patient did not report any symptoms of cortisol deficiency. Another four patients had baseline cortisol results above 400 nmol/L and none of these patients had any symptoms of glucocorticoid insufficiency. As such these patients were thought overall to be ACTH sufficient. 11 patients completed the confirmatory SST, and all achieved a peak cortisol response of greater than 450 nmol/L. Two patients did not attend their follow up SST (Fig. 1).

### Arginine stimulation test

Following the initial GST and confirmatory testing of the ACTH axis, 26 patients were diagnosed with isolated GHD and therefore were required to undergo confirmatory testing of the somatotrophic axis. Six of these patients had QoL-AGHDA score of less than 11 and thus did not meet NICE guidelines for GH replacement [28]. Five of these patients declined the confirmatory AST. Therefore, 21 patients underwent the AST and this confirmed isolated GHD in 13 patients (Fig. 1).

After confirmatory testing, the total number of patients with GHD in our cohort was 14 (thirteen patients with isolated GHD and one patient with GHD combined with hypogonadism). Isolated gonadotrophin deficiency was noted in three further patients. Assuming that the two patients who did not attend follow up SST were ACTH deficient (given that we were not able to confirm this), the prevalence of hypopituitarism in our cohort is 19%.

### Factors related to GHD

**Fig. 2:**
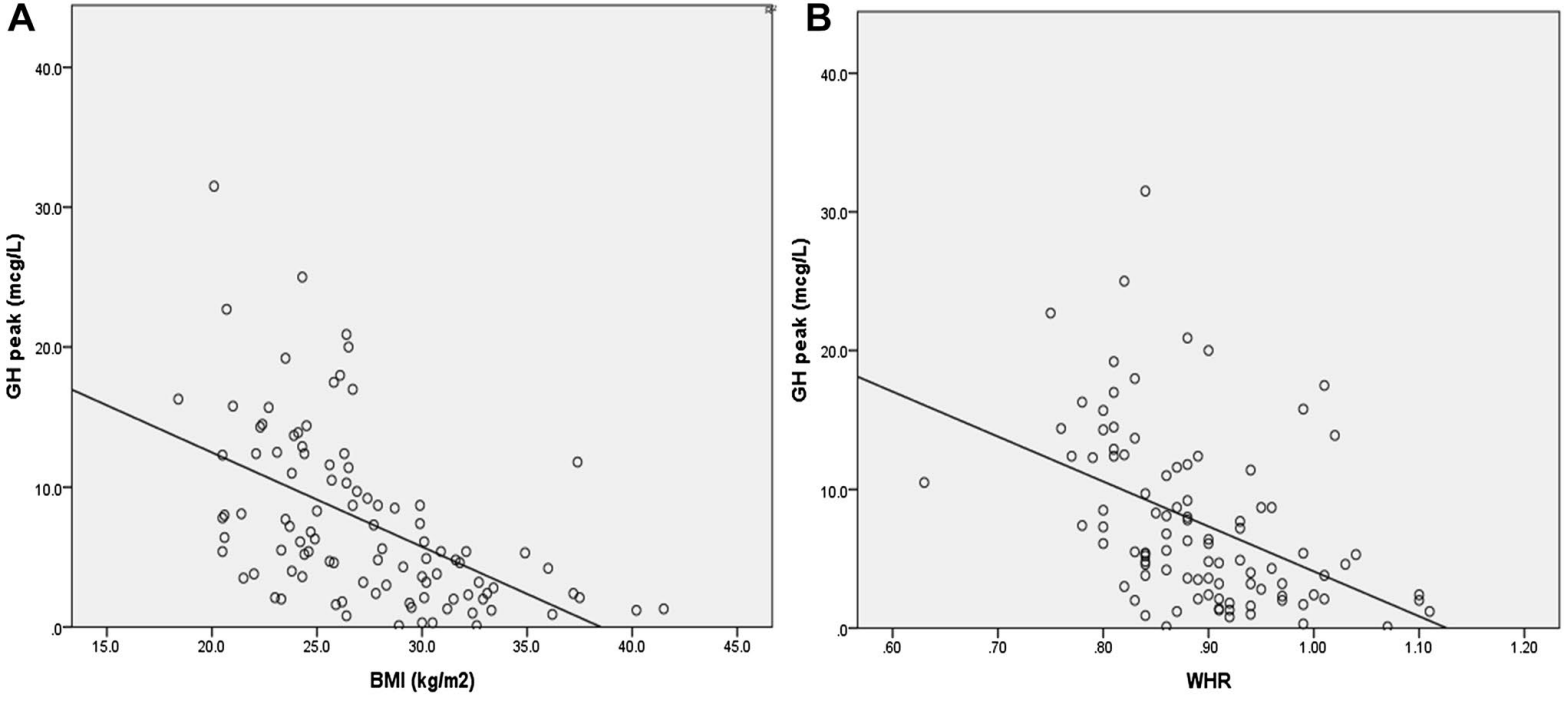
Correlation between peak GH on GST with BMI and WHR. **a** Peak GH response on GST versus BMI in all patients in our cohort, R = −0.519 (p < 0.001), **b** Peak GH response on GST versus waist to hip ratio (WHR), R = −0.434 (p < 0.001)

On univariate analysis, the differences in body weight, BMI and WHR between patients with GHD compared to patients without GHD were significant (Table 3). There was a negative correlation between peak GH level on GST and the patient’s BMI (R = −0.52; p < 0.0001) and the WHR in our cohort (R = −0.43; p < 0.0001; Fig. 2).

**Table 3:** Comparison between patients with confirmed GHD and those without GHD

	GHD (n = 14)	Not GHD (n = 86)	p-value
M/F	7/7	24/61	0.13^a^
Mean weight (kg)	88.3 ± 13.3	72.4 ± 14.2	<0.0001^b^
Mean BMI (kg/m2)	31.9 ± 3.9	26.6 ± 4.5	<0.0001^b^
Mean WHR	0.94 ± 0.07	0.88 ± 0.08	0.008^b^
Mean age at presentation (years)	52 ± 10	53 ± 10	0.73^b^
Mean time to screening (months)	34 ± 18	48 ± 32	0.25^c^
Mean age at screening (years)	55 ± 10	57 ± 10	0.66^b^
Procedure			0.11^d^
None	3	11	
Endovascular coiling	7	60	
Clipping	2	13	
Multiple endovascular coiling	2	2	
Site of aneurysm			0.61^d^
Anterior circulation			
Anterior communicating artery	3	24	
Middle cerebral artery	2	17	
Posterior communicating artery	3	15	
Internal carotid artery	0	7	
Pericallosal artery	0	1	
Posterior circulation			
Basilar artery	1	5	
Posterior inferior cerebellar artery	0	3	
Vertebral artery	0	1	
Perimesencephalic	3	11	
Multiple aneurysms	2	2	
WFNS			0.58^d^
1	10	59	
2	3	10	
3	0	4	
4	1	2	
5	0	6	
Fisher			0.57^d^
1	3	10	
2	2	13	
3	1	10	
4	4	35	
GOS			0.03^a^
4	5	10	
5	9	76	

Fisher grading on admission was not available for eight patients. WFNS grading on admission was not available for four patients

^a^Chi-square test

^b^ *t* test

^c^Mann–Whitney *U* test

^d^Fisher exact test

On univariate analysis there was no significant difference between the patients with GHD and those without GHD, with respect to the mean age of onset of SAH (p = 0.73), the age (p = 0.66) and time to screening post SAH (p = 0.25), the GCS at presentation (p = 0.52), WFNS grade (p = 0.58), site of aneurysm (p = 0.61), Fisher grade of SAH (p = 0.57), EVD insertion (p = 0.26), occurrence of vasospasm (p = 0.33) and treatment modality (p = 0.11; Table 3). Patients with GHD had worse GOS scores at screening than those without GHD (p = 0.03; Table 3).

Logistic regression was carried out to assess how the covariates of BMI, WHR, ADGHA score, gender, age at screening and hydrocephalus were associated with GHD. After running our models, all of which included BMI, gender, WHR, age, QoL-AGHDA score and GOS as covariates, we found the following to be positively associated with GHD: BMI (OR 1.527, 95% CI 1.17–1.994), AGHDA score (OR 1.38, 95% CI 1.102–1.737) and hydrocephalus (OR 7.671, 95% CI 1.139–51.68). In spite of the small number of patients with GHD this model remained resilient. However the interpretation of these results should be done cautiously. For example the unfeasibly high upper confidence interval of hydrocephalus suggests an odds ratio of 51.68, and is unlikely to reflect the truth.

## Discussion

This is a large study assessing endocrine function in SAH patients with dynamic testing of pituitary function. After the initial GST, GHD was detected in 27% of patients which is consistent with other studies employing this test [19, 29]. Additional testing with the arginine stimulation test, reduced prevalence of GHD detected in our cohort to 14% and the prevalence of hypopituitarism to 19%. This is consistent with more recent studies that incorporated confirmatory testing [18–20]. This study also confirms that isolated GHD is the most common pituitary hormone deficiency post subarachnoid haemorrhage.

The retrospective method of recruitment in this study is likely to favour patients with better clinical outcomes and less severe SAH. This is reflected by the high proportion of survivors with good WFNS grade and GOS score at screening in our cohort and may not truly reflect the risk of hypopituitarism after severe SAH. However, this preponderance of patients with mild-moderate clinical severity and high proportion of treatment via the endovascular route is similar to other modern cohorts [19–21]. It is noted however, that the radiological severity as measured by the Fisher grade in our cohort is less severe than other studies and this might confound our results. Perimesencephalic-type SAH is associated with good clinical outcome [30] and the inclusion of these patients (14%) in our analysis may additionally contribute to the low prevalence of GHD in our cohort.

As it was not compulsory for participants to undergo confirmatory testing of the somatotrophic axis if the QoL-AGHDA score was <11, it is possible that the prevalence of isolated GHD is slightly underestimated in our cohort. It is understandable that some participants who did not meet NICE criteria for GHR were reluctant to re-attend our centre for a second dynamic test, as they were not eligible for GHR. Additional factors such as poor mental and physical health were contributory factors for the poor compliance in this regard. However this does highlight one of the difficulties in conducting a ‘real life’ clinical study in this patient population and should be considered when planning further studies involving this patient group.

The variation in reported prevalence of hypopituitarism and GHD in the literature is most likely reflective of the heterogeneous endocrine tests used to diagnose hormonal deficiency, different time points of endocrine assessment and varying thresholds for defining GHD [23, 31, 32]. Indeed, several of previous studies relied solely on low serum IGF-1 concentrations to diagnose GHD [13, 15, 33, 34], in spite of its limited diagnostic accuracy [35–37]. Additionally, even though isolated GHD is consistently reported as the most common deficiency post SAH, few studies confirm this with a second dynamic test [19–21]. The potential to misdiagnose isolated GHD on a single dynamic test is well established [38] and as such it is likely that studies that have only relied on a single test may over-estimate the prevalence of GHD [14, 39]. Studies that have employed dynamic pituitary tests and subsequent confirmatory testing report a lower prevalence of GHD of between 0 and 13.3% [19–21]. Therefore the size of our study and the use of two different dynamic tests to confirm GHD strengthens the validity of our results.

The difference in terms of weight, BMI and WHR between patients with confirmed GHD and those with adequate GH response in our cohort, was statistically significant. Although it has been demonstrated that GHD in adults is associated with increased weight, body fat and central adiposity [40, 41], it is unclear if our findings are truly reflective of this and therefore causality cannot be assumed. The GST has been shown to overestimate GHD in overweight adults with no known pituitary disease [42]. We have demonstrated an inverse correlation between the peak GH on GST and both BMI and WHR. Obesity is recognised as a confounder of GH response [42–44]. The mechanism by which glucagon stimulates GH release is unclear, however it is not unreasonable to postulate that it may be affected by the metabolic consequences associated with weight gain and central adiposity.

Even other stimulatory tests, such as the insulin tolerance test (ITT) and growth hormone releasing hormone (GHRH)-arginine stimulation test, show that GH response is BMI dependent [44, 45]. It has even been suggested that waist circumference corrected cut-offs for GH peaks should be established [37] given that serum GH levels correlate inversely with WHR and abdominal fat in adults with no pituitary disease [46]. Diagnosing GHD in the presence of obesity can therefore be very challenging, and an appreciation of this is vital to ensure that results are interpreted with caution.

Interestingly, no difference in BMI between patients who were hypopituitary and those with normal pituitary function was demonstrated by Gardner et al., who employed GHRH-arginine stimulation test with BMI-specific cut-offs in their cohort [19]. It is noteworthy that studies that incorporate the GHRH-arginine stimulation test with its validated BMI-specific cut-offs report the prevalence of GHD to be only be as high as 10% [18, 19, 21]. Kronvall et al. 2014, using the GHRH-arginine test reported a 7% prevalence of GHD at 3–6 months and 25% at 12–24 months testing [47]. However it is noted that at 12–24 months, patients were tested with either the ITT or GHRH-arginine and this may account for the higher prevalence of GHD detected at that time point. There are studies that have reported higher frequency of GHD even when GHRH-arginine stimulation test was used, however these studies did not incorporate current validated BMI specific cut-offs when diagnosing GHD [16, 39]. Therefore it is possible that GHD is also overestimated in our cohort, due to the limitations mentioned above. Further assessment of this cohort with a GHRH-arginine stimulation tests is recommended.

Like other studies, there was no association between development of GHD and clinical or radiological severity of SAH, GCS at presentation, age at presentation, treatment modality or the presence of vasospasm [2, 15, 16, 33] in this cohort.

Given the lack of clinical predictors of GHD in this patient population consensus guidelines recommend endocrine screening in all SAH survivors in whom there is an intention to treat [48, 49]. However such a recommendation may have a significant impact on clinical practice. Routine screening for endocrine dysfunction in all SAH survivors with dynamic pituitary testing incurs a significant financial, logistic and work force requirement. Furthermore, such a screening process will subject a large number of patients to the unpleasant and on occasion detrimental side-effects associated with dynamic pituitary testing. Importantly, the value of detecting GHD in this patient population remains unclear, given that data regarding the impact or benefit of Growth Hormone Replacement in SAH survivors is scant. Lastly, uncertainty exists as to whether development of pituitary dysfunction after SAH is permanent. Several authors including a recent meta-analysis, have demonstrated that the prevalence of pituitary dysfunction including that of the somatotrophic axis, can change with time [14, 19, 22, 23, 29, 39, 47, 50]. As such it remains unclear whether pituitary function should be continually re-assessed in SAH patients, and if so, at what time points.

## Conclusion

The prevalence of GHD in our cohort was 14%, when dynamic pituitary testing and confirmatory testing was employed. The varied prevalence reported in the literature is likely due to heterogeneous testing methods. Even though there was a significant difference in terms of BMI and WHR in patients with and without GHD, this may be due to the testing methodology employed. Further testing with protocols that incorporate BMI-specific cut-offs are planned. Even though GHD is associated with worse quality of life as measured by the QoL-AGHDA score, there are no good clinical predictors of GHD. Although it is recommended that all SAH survivors be screened for endocrine dysfunction, this may not be always be feasible and has a substantial impact on resources. Further guidance is required as to which patients to assess, type of endocrine tests to use, timing of patient assessment and importantly which patients would derive clinical benefit from growth hormone testing and subsequently hormonal replacement.

## Appendix Clinical protocols for dynamic tests used

### Glucagon stimulation test

- After an overnight fast(from midnight onwards-water allowed) an intravenous cannula is inserted and allowed to rest for 30 min
- Baseline sample for of GH, IGF-1, testosterone/estradiol, LH, FSH, cortisol, ACTH, fT4, TSH and prolactin is drawn.
- Glucagon (GlucaGen, Novonordisk Ltd) is administered subcutaneously at a dose of 1 mg (1.5 mg for patients >100 kgs).
- Blood samples for GH and cortisol are taken at 90, 120, 150, 180, 210 and 240 min.

### Arginine stimulation test

- After an overnight fast as above, intravenous cannulae are inserted into both forearms and are allowed to rest for 30 min
- A baseline blood sample is taken at 0 min for GH
- 30 g arginine is infused over 30 min (150 mls of 20% l-arginine hydrochloride (Stockport Pharmaceuticals, UK))
- Blood samples for GH are taken at 30, 60, 90 and 120 min.

### Short synacthen test

- Following an overnight fast as above, an indwelling peripheral venous cannula is inserted and allowed to rest for 30 min
- Baseline blood sample for cortisol measurement is drawn
- Intramuscular injection of 250 µg of synacthen (Tetracosactide, Questcor Operations, UK) is administered
- Blood samples for cortisol are drawn at 30 and 60 min.

## References

[1] Quinn AC, Bhargava D, Al-Tamimi YZ, Clark MJ, Ross SA, Tennant A (2014). Self-perceived health status following aneurysmal subarachnoid haemorrhage: a cohort study. BMJ Open.

[2] Kreitschmann-Andermahr I, Hoff C, Saller B, Niggemeier S, Pruemper S, Hutter BO (2004). Prevalence of pituitary deficiency in patients after aneurysmal subarachnoid hemorrhage. J Clin Endocrinol Metab.

[3] Rinkel GJE, Algra A (2011). Long-term outcomes of patients with aneurysmal subarachnoid haemorrhage. Lancet Neurol.

[4] Visser-Meily JMA, Rhebergen ML, Rinkel GJE, van Zandvoort MJ, Post MWM (2009). Long-term health-related quality of life after aneurysmal subarachnoid hemorrhage relationship with psychological symptoms and personality characteristics. Stroke.

[5] Wong GKC, Poon WS, Boet R, Chan MTV, Gin T, Ng SCP (2011). Health-related quality of life after aneurysmal subarachnoid hemorrhage: profile and clinical factors. Neurosurgery.

[6] Ronne-Engstrom E, Enblad P, Lundstrom E (2013). Health-related quality of life at median 12 months after aneurysmal subarachnoid hemorrhage, measured with EuroQoL-5D. Acta Neurochir.

[7] Tjahjadi M, Heinen C, Koenig R, Rickels E, Wirtz CR, Woischneck D (2013). Health-related quality of life after spontaneous subarachnoid hemorrhage measured in a recent patient population. World Neurosurg.

[8] Noble AJ, Baisch S, Mendelow AD, Allen L, Kane P, Schenk T (2008). Posttraumatic stress disorder explains reduced quality of life in subarachnoid hemorrhage patients in both the short and long term. Neurosurgery.

[9] Scott RB, Eccles F, Molyneux AJ, Kerr RSC, Rothwell PM, Carpenter K (2010). Improved cognitive outcomes with endovascular coiling of ruptured intracranial aneurysms neuropsychological outcomes from the international subarachnoid aneurysm trial (ISAT). Stroke.

[10] Kronvall E, Sonesson B, Valdemarsson S, Siemund R, Säveland H, Nilsson OG (2016). Reduced quality of life in patients with pituitary dysfunction after aneurysmal subarachnoid hemorrhage: a prospective longitudinal study. World Neurosurg.

[11] Khajeh L, Ribbers GM, Heijenbrok-Kal MH, Blijdorp K, Dippel DWJ, Sneekes EM (2016). The effect of hypopituitarism on fatigue after subarachnoid hemorrhage. Eur J Neurol.

[12] Kelly DF, Gonzalo IT, Cohan P, Berman N, Swerdloff R, Wang C (2000). Hypopituitarism following traumatic brain injury and aneurysmal subarachnoid hemorrhage: a preliminary report. J Neurosurg.

[13] Jovanovic V, Pekic S, Stojanovic M, Tasic G, Djurovic B, Soldatovic I (2010). Neuroendocrine dysfunction in patients recovering from subarachnoid hemorrhage. Hormones.

[14] Tanriverdi F, Dagli AT, Karaca Z, Unluhizarci K, Selcuklu A, Casanueva FF (2007). High risk of pituitary dysfunction due to aneurysmal subarachnoid haemorrhage: a prospective investigation of anterior pituitary function in the acute phase and 12 months after the event. Clin Endocrinol.

[15] Dimopoulou I, Kouyialis AT, Tzanella M, Armaganidis A, Thalassinos N, Sakas DE (2004). High incidence of neuroendocrine dysfunction in long-term survivors of aneurysmal subarachnoid hemorrhage. Stroke.

[16] Aimaretti G, Ambrosio MR, Di Somma C, Fusco A, Cannavo S, Gasperi M (2004). Traumatic brain injury and subarachnoid haemorrhage are conditions at high risk for hypopituitarism: screening study at 3 months after the brain injury. Clin Endocrinol.

[17] Lammert A, Bode H, Hammes HP, Birck R, Fatar M, Zohsel K (2012). Aneurysmal subarachnoid hemorrhage (aSAH) results in low prevalence of neuro-endocrine dysfunction and NOT deficiency. Pituitary.

[18] Kronvall E, Valdemarsson S, Saveland H, Nilsson OG (2014). Pituitary dysfunction after aneurysmal subarachnoid hemorrhage is associated with impaired early outcome. World Neurosurg.

[19] Gardner CJ, Javadpour M, Stoneley C, Purthuran M, Biswas S, Daousi C (2013). Low prevalence of hypopituitarism after subarachnoid haemorrhage using confirmatory testing and with BMI-specific GH cut-off levels. Eur J Endocrinol.

[20] Hannon MJ, Behan LA, O’Brien MM, Tormey W, Javadpour M, Sherlock M (2014). Chronic hypopituitarism is uncommon in survivors of aneurysmal subarachnoid haemorrhage. Clin Endocrinol.

[21] Klose M, Brennum J, Poulsgaard L, Kosteljanetz M, Wagner A, Feldt-Rasmussen U (2010). Hypopituitarism is uncommon after aneurysmal subarachnoid haemorrhage. Clin Endocrinol.

[22] Khajeh L, Blijdorp K, Heijenbrok-Kal MH, Sneekes EM, van den Berg-Emons HJG, van der Lely AJ (2014). Pituitary dysfunction after aneurysmal subarachnoid haemorrhage: course and clinical predictors the HIPS study. J Neurol Neurosurg Psychiatry.

[23] Can A, Gross BA, Smith TR, Dammers R, Dirven CMF, Woodmansee WW (2016). Pituitary dysfunction after aneurysmal subarachnoid hemorrhage: a systematic review and meta-analysis. Neurosurgery.

[24] Drake CG (1988). Report of World-Federation-of-Neurological-Surgeons Committee on a Universal subarachnoid hemorrhage Grading Scale. J Neurosurg.

[25] Fisher CM, Kistler JP, Davis JM (1980). Relation of cerebral vasospasm to subarachnoid hemorrhage visualized by computerized tomographic scanning. Neurosurgery.

[26] Jennett B, Bond M (1975). Assessment of outcome after severe brain-damage - practical scale. The Lancet.

[27] NICE. Quality of life. Assessment of GH deficiency in adults. https://www.nice.org.uk/guidance/ta64/documents/questionnaire-quality-of-life-assessment-of-deficiency-in-adults2.

[28] (NICE) NIoHaCE (2003) Human growth hormone (somatropin) in adults with growth hormone deficiency. https://www.nice.org.uk/guidance/ta64.

[29] Karaca Z, Tanriverdi F, Dagli AT, Selcuklu A, Casanueva FF, Unluhizarci K (2013). Three years prospective investigation of pituitary functions following subarachnoid haemorrhage. Pituitary.

[30] Flaherty ML, Haverbusch M, Kissela B, Kleindorfer D, Schneider A, Sekar P (2005). Perimesencephalic subarachnoid hemorrhage: incidence, risk factors, and outcome. J Stroke Cerebrovasc Dis.

[31] Ioachimescu AG, Barrow DL (2015). Subarachnoid hemorrhage and the pituitary. World Neurosurg.

[32] Schneider HJ, Kreitschmann-Andermahr I, Ghigo E, Stalla GK, Agha A (2007). Hypothalamopituitary dysfunction following traumatic brain injury and aneurysmal subarachnoid hemorrhage: a systematic review. Jama.

[33] Parenti G, Cecchi PC, Ragghianti B, Schwarz A, Ammannati F, Mennonna P (2011). Evaluation of the anterior pituitary function in the acute phase after spontaneous subarachnoid hemorrhage. J Endocrinol Invest.

[34] Dutta P, Mukherjee KK, Chaudhary PK, Masoodi SR, Anand S, Pathak A (2012). Pituitary dysfunction in survivors of spontaneous subarachnoid hemorrhage of anterior communicating artery and middle cerebral artery aneurysms: a comparative study. Neurology India.

[35] Svensson J, Johannsson G, Bengtsson BA (1997). Insulin-like growth factor-I in growth hormone-deficient adults: Relationship to population-based normal values, body composition and insulin tolerance test. Clin Endocrinol.

[36] Toogood AA, Jones J, O’Neill PA, Thorner MO, Shalet SM (1998). The diagnosis of severe growth hormone deficiency in elderly patients with hypothalamic-pituitary disease. Clin Endocrinol.

[37] Andersen M (2015). The robustness of diagnostic tests for GH deficiency in adults. Growth Horm IGF Res.

[38] Lissett CA, Thompson EGE, Rahlm A, Brennan BMD, Shalet SM (1999). How many tests are required to diagnose growth hormone (GH) deficiency in adults?. Clin Endocrinol.

[39] Aimaretti G, Ambrosio MR, Di Somma C, Gasperi M, Cannavo S, Scaroni C (2005). Residual pituitary function after brain injury-induced hypopituitarism: a prospective 12-month study. J Clin Endocrinol Metab.

[40] Bengtsson BA (1998). Untreated growth hormone deficiency explains premature mortality in patients with hypopituitarism. Growth Horm IGF Res.

[41] Hoffman AR, Kuntze JE, Baptista J, Baum HBA, Baumann GP, Biller BMK (2004). Growth hormone (GH) replacement therapy in adult-onset GH deficiency: effects on body composition in men and women in a double-blind, randomized, placebo-controlled trial. J Clin Endocrinol Metab.

[42] Dichtel LE, Yuen KCJ, Bredella MA, Gerweck AV, Russell BM, Riccio AD (2014). Overweight/obese adults with pituitary disorders require lower peak growth hormone cutoff values on glucagon stimulation testing to avoid overdiagnosis of growth hormone deficiency. J Clin Endocrinol Metab.

[43] Klose M, Feldt-Rasmussen U (2015). Hypopituitarism in traumatic brain injury-a critical note. J Clin Med.

[44] Corneli G, Di Somma C, Baldelli R, Rovere S, Gasco V, Croce CG (2005). The cut-off limits of the GH response to GH-releasing hormone-arginine test related to body mass index. Eur J Endocrinol.

[45] Biller BMK, Samuels MH, Zagar A, Cook DM, Arafah BM, Bonert V (2002). Sensitivity and specificity of six tests for the diagnosis of adult GH deficiency. J Clin Endocrinol Metab.

[46] Vahl N, Jorgensen JOL, Skjaerbaek C, Veldhuis JD, Orskov H, Christiansen JS (1997). Abdominal adiposity rather than age and sex predicts mass and regularity of GH secretion in healthy adults. Am J Physiol-Endocrinol Metab.

[47] Kronvall E, Sonesson B, Valdemarsson S, Siemund R, Saveland H, Nilsson OG (2016). Reduced quality of life in patients with pituitary dysfunction after aneurysmal subarachnoid hemorrhage: a prospective longitudinal study. World Neurosurg.

[48] Ho KKY, Works GHDC (2007). Consensus guidelines for the diagnosis and treatment of adults with GH deficiency II: a statement of the GH research society in association with the European Society for Pediatric Endocrinology, Lawson Wilkins Society, European Society of Endocrinology, Japan Endocrine Society, and Endocrine Society of Australia. Eur J Endocrinol.

[49] Gross BA, Laws ER (2015). Pituitary dysfunction after aneurysmal subarachnoid hemorrhage. World Neurosurg.

[50] Kopczak A, Krewer C, Schneider M, Kreitschmann-Andermahr I, Schneider HJ, Stalla GK (2015). The development of neuroendocrine disturbances over time: longitudinal findings in patients after traumatic brain injury and subarachnoid hemorrhage. Int J Mol Sci.

